# NHERF1 inhibits beta-catenin-mediated proliferation of cervical cancer cells through suppression of alpha-actinin-4 expression

**DOI:** 10.1038/s41419-018-0711-x

**Published:** 2018-06-04

**Authors:** Qiqi Wang, Qiong Qin, Ran Song, Chunjuan Zhao, Hua Liu, Ying Yang, Siyu Gu, Deshan Zhou, Junqi He

**Affiliations:** 1 Department of Biochemistry and Molecular Biology, Capital Medical University, Beijing, China; 2 Beijing Key Laboratory for Tumor Invasion and Metastasis, Beijing, China; 3 Core Facilities Center, Capital Medical University, Beijing, China; 4 Department of Histology and Embryology, Capital Medical University, Beijing, China

## Abstract

Cervical cancer is one of the most lethal types of cancer in female. Aberrant activation of Wnt/β-catenin signaling pathway has been found to be involved in cervical cancer development and progression, whereas the underlying molecular mechanisms remain poorly understood. The present study showed that NHERF1 was a novel gene associated with both cell proliferation and Wnt signaling pathway in cervical cancer by analysis of differential gene expression and gene cluster for the cervical cancer specimens from GEO data sets. It was further demonstrated in cellular study that NHERF1 inhibition of cervical cancer cell proliferation through Wnt/β-catenin signaling was dependent on α-actinin-4 (ACTN4) expression. A negative association between NHERF1 expression and levels of ACTN4 and β-catenin was found in mouse xenograft model and cervical cancer specimens. Low levels of NHERF1 in cervical cancer specimens were found to associate with activation of cell proliferation and Wnt/β-catenin signaling by gene set enrichment analysis, and also were an independent predictive factor for worse prognosis of cervical cancer patients by Cox regression analysis. These findings demonstrate that NHERF1 inhibits Wnt signaling-mediated proliferation of cervical cancer via suppression of ACTN4, and NHERF1 downregulation may contribute to the progression of cervical cancer. These findings may also shed some lights for understanding the underlying mechanisms of cisplatin resistance and worse prognosis of HPV-inactive cervical cancer patients.

## Facts

- Expression level of NHERF1 was reduced significantly in cervical cancer (CC) tissues.
- NHERF1 inhibited CC cell proliferation via attenuation of Wnt/β-catenin signaling.
- NHERF1 attenuated β-catenin expression via suppression of α-actinin-4 expression.
- Downregulation of NHERF1 was involved in the development and progression of CC and may serve as a potential predictor of prognosis and cisplatin response for CC patients.

## Introduction

Cervical cancer is the fourth most common cancer in women worldwide with 500,000 new cases and 233,000 deaths per year, and the second leading cause of cancer death for women living in developing countries^1^. High-risk human papilloma virus (HR-HPV), which produces oncogenic types of HPV proteins, is strongly correlated with cervical cancer. However, only a small ratio of HPV-infected patients develop cancer, and factors such as genetic and epigenetic changes acting synergistically have been implicated to the progression from cervical precancerous lesions to cervical cancer^2^. Therefore, extensive studies of the molecular mechanisms that modulate the progression of cervical cancer are crucial for the enabling of early diagnosis and effective treatment for cervical cancer.

Uncontrolled cellular proliferation caused by dysregulation of several major molecular signaling pathways is a major feature of cervical epithelial malignancy^3,4^. Overactivation of MAPK/ERK or PI3K/Akt pathways^5,6^ and their components, such as EGFR^5,7,8^ and Ras^9^, was observed in cervical cancer and correlated to the neoplastic progression of cervical neoplasia. In the past decades, increasing evidences suggested that aberrant activation of Wingless-type (Wnt)/β-catenin pathway plays major roles during the multistep processes, including cell proliferation and metastasis in cervical cancer carcinogenesis and progression^10,11^. HR-HPV is a key factor during cervical cancer development, and hyperactivation of Wnt pathway has been demonstrated in HPV-associated cancers^12,13^. The activation of Wnt signaling induced by HPV oncoproteins, such as E6 and E7 proteins, have been indicated to contribute to the onset, progression, and maintenance of cancerous transformed cells in vitro models and in transgenic mice^12–15^. Recently, the study has shown that Wnt/β-catenin signaling was also implicated in the carcinogenesis and propagation of HPV-negative or low E6/E7-expressed cervical cancer^16^. Lines of evidences indicated that induction of apoptosis and suppression of tumor growth, cell motility, invasion, and angiogenesis in cervical cancer could be achieved via inhibition of Wnt signaling^17,18^. These studies suggest a significant role of Wnt/β-catenin signaling during cervical cancer development regardless of HPV status.

Beta-catenin acts as the central factor in canonical Wnt signaling. When Wnt ligand is presented, accumulated β-catenin entries into the nucleus to activate gene transcription, such as c-Myc, TCF-1, and Cyclin D1, in controlling cellular processes such as proliferation, differentiation, and migration^19^. High expression levels of β-catenin were observed during cancer progression in cervical cancer biopsies^20^ and have been considered as a poor prognostic factor for cervical cancer^21^. Although mutations in several components, including β-catenin of the Wnt pathway, have been verified in various types of cancer^22^, such as colorectal carcinoma^23^, mutation of β-catenin was rarely detected in cervical cancer^14^. Thus, our understanding of the molecular mechanisms underlying aberrant activation of Wnt/β-catenin signaling in cervical cancer is still incomplete.

In the present study, identification for differential gene expression between tumor and normal tissues using the available mRNA data profiles of cervical cancer specimens from GEO data sets combined with DAVID (The Database for Annotation, Visualization and Integrated Discovery) analysis was applied for the screening of genes associated with both cell proliferation and Wnt pathway. Among the 1615 differentially expressed genes, Na^+^/H^+^ exchanger regulatory factor 1 (NHERF1, also called ezrin-radixin-moesin-binding phosphoprotein 50/EBP50), were a novel gene which was downregulated and associated with cell proliferation and Wnt pathway in cervical cancer specimens. NHERF1 was further demonstrated to retard cell proliferation with the attenuation of Wnt/β-catenin pathway activation of cervical cancer cells in vivo and in vitro through suppression of α-actinin-4 (ACTN4) expression level. Downregulation of NHERF1 was verified to be correlated with activation of proliferation, and Wnt/β-catenin signaling and adverse prognosis in cervical cancer. These data reveal a novel tumor-suppressive role of NHERF1 and provide new insights into the development and progression of cervical cancer.

## Results

### *NHERF1* is a novel downregulated gene correlated with cell proliferation and Wnt signaling in cervical cancer

**Fig. 1:**
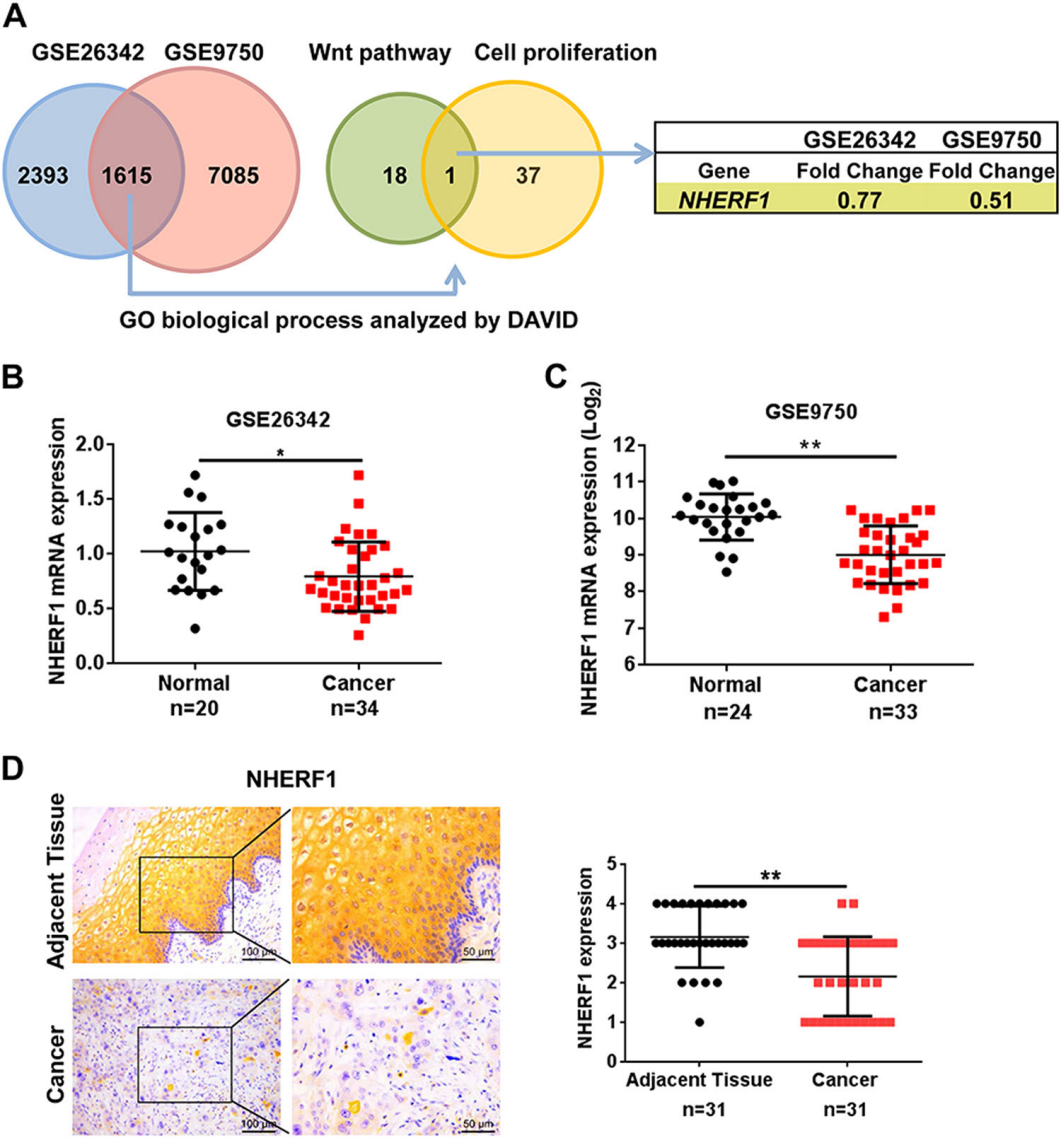
NHERF1 is significantly downregulated and correlated with cell proliferation and Wnt signaling in cervical cancer. **a** The differentially expressed genes between the cervical cancer specimens and normal cervix tissues were identified by significance analysis of microarrays, and 1615 differential expression genes were found both in GSE26342 and GSE9750 data sets. The median FDR < 0.05 was used as a cutoff value. Analysis of the differentially expressed genes was performed with the DAVID analysis of gene ontology. NHERF1 was associated with negative regulation of cell proliferation and Wnt pathways. **b**, **c** Scatter plots of relative NHERF1 mRNA levels in cervical cancer specimens and their adjacent tissues, the data were obtained from GSE26342 (**b**) and GSE9750 (**c**) (*t* test, *p < 0.05, **p < 0.01, error bars represent mean ± s.d.). **d** The NHERF1 immunohistochemistry staining images of a tissue microarray with 31 paired human cervical cancer specimens and adjacent normal tissues. The values of NHERF1 were quantified by grading method (nonparametric test, Mann–Whitney test, **p *<* 0.01, error bars represent mean ± s.d.)

To identify differential-expressed genes in cervical cancer, we compared the gene expression profiles between cervical cancer and normal cervix tissues through microarray data obtained from GEO database. The data sets of GSE26342 and GSE9750 were analyzed by significance analysis of microarrays with the median FDR < 0.05 as a cutoff value^24^. A total of 1615 genes were identified that differed significantly in expression in both data sets. Functional clustering analysis of these 1615 genes revealed that 19 genes were involved in Wnt pathway, and 38 genes participated in negative regulation of cell proliferation. *NHERF1* was the only gene associated with both cell proliferation and Wnt signaling (Fig. 1a). To verify these findings, the levels of NHERF1 mRNA in cervical cancer and their adjacent tissues were analyzed, and the results showed that NHERF1 mRNA was significantly decreased in the above two independent data sets (Fig. 1b, c). To further analyze the protein levels of NHERF1 in cervical cancer, a tissue microarray containing 31 paired cervical cancer and adjacent tissue specimens were used to analyze the expression level of NHERF1. The protein levels of NHERF1 were also robustly reduced in cervical cancer specimens (Fig. 1d). Therefore, these findings suggest a tumor-suppressive role of NHERF1 in cervical cancer. Wnt signaling and cell proliferation associated with downregulation of NHERF1 might contribute to cervical cancer development and progression.

### NHERF1 inhibits cervical cancer cell proliferation in vitro

**Fig. 2:**
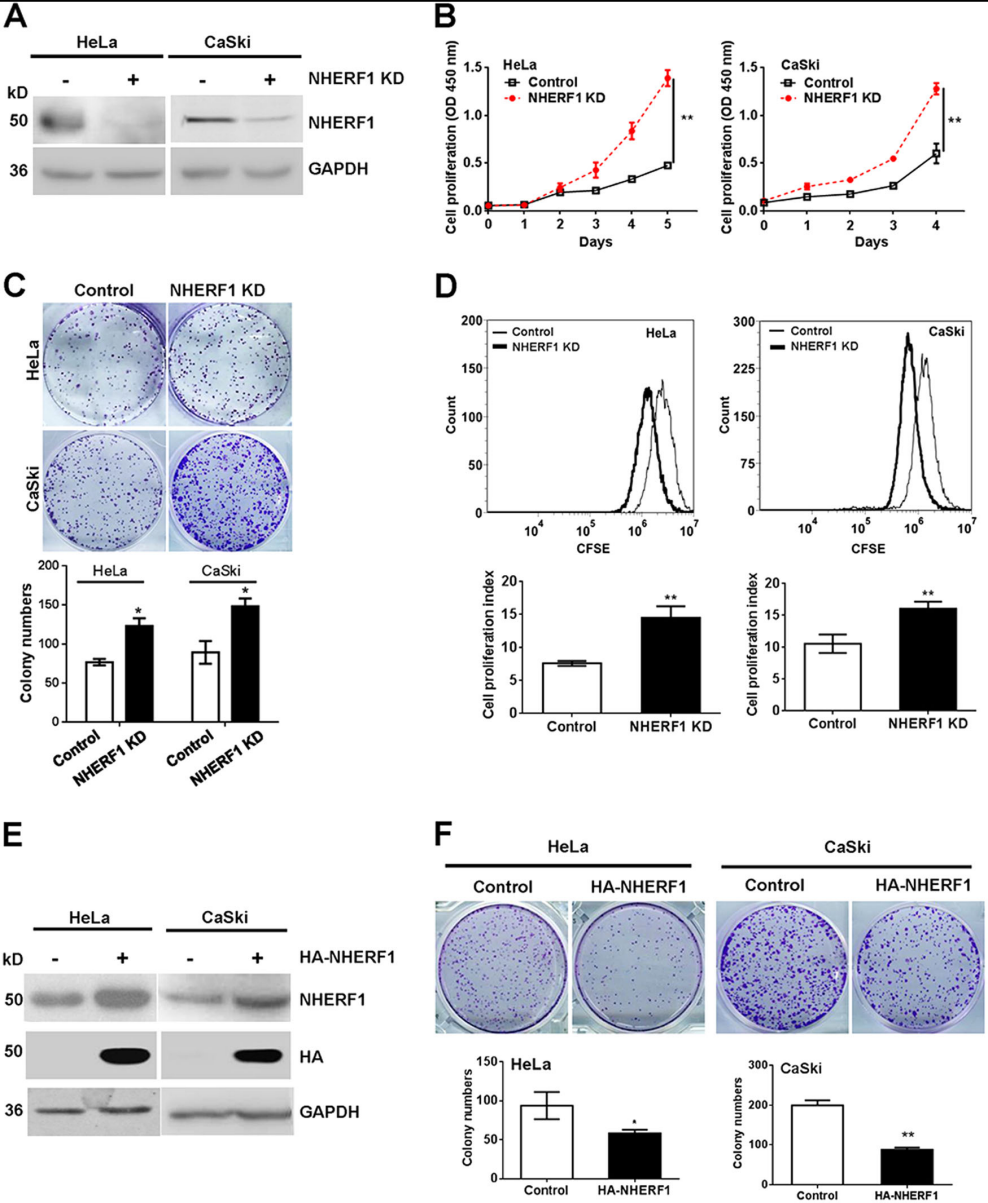
NHERF1 inhibits cervical cancer cell proliferation. **a** Knockdown of NHERF1 expression in cervical cancer cells was verified by immunoblotting analysis. HeLa cells were stably transfected with shNHERF1 constructs (HeLa-NHERF1-KD), and CaSki cells were transiently transfected with NHERF1 siRNAs (CaSki-NHERF1-KD). **b** Knockdown of NHERF1 enhanced proliferation of cervical cancer cells. Proliferation of HeLa-NHERF1-KD, CaSki-NHERF1-KD, and their control cells was detected by CCK-8 at the indicated time points (repeated-measures analysis of variance, **p *<* 0.01, error bars represent mean ± s.d., *n* = 3). **c** Knockdown of NHERF1 enhanced the colony formation of cervical cancer cells. Colony formation was monitored in HeLa or CaSki cells for 7 days. Top panel: Representative photographs of the clonogenicity. Bottom panel: Quantification of the colony formation efficiency (*t* test, *p < 0.05, error bars represent mean ± s.d., *n* = 3). **d** Inhibition of NHERF1 expression enhanced cell proliferation of cervical cancer cells by CFSE assay (*t* test, **p *<* 0.01, error bars represent mean ± s.d., *n* = 3). Cells were stained with CFSE and analyzed following the protocol as described in the “Methods”. **e** Overexpression of NHERF1 in cervical cancer cells was verified by immunoblotting analysis. HeLa and CaSki cells were transiently transfected with NHERF1 constructs, respectively, and expression of NHERF1 was verified by western blotting. **f** Exogenous NHERF1 expression inhibited the colony formation of cervical cancer cells. Colony formation was monitored in HeLa or CaSki cells for 7 days. Top panel: Representative photographs of the clonogenicity. Bottom panel: Quantification of the efficiency of colony formation (*t* test, *p *<* 0.05, error bars represent mean ± s.d., *n* = 3). Cells proliferation was detected by CCK-8 assay at the indicated time points (repeated-measures analysis of variance, **p *<* 0.01, error bars represent mean ± s.d., *n* = 3)

The mRNA levels of NHERF1 of 14 cervical cancer cell lines were analyzed in GSE9750 or GSE89657 data set. HeLa (cervical adenocarcinoma cell line with high level of NHERF1) and CaSki (cervical squamous cell carcinoma with low level of NHERF1) cells were chosen in this study (Fig. S1). To investigate the roles of NHERF1 in cervical cancer cell proliferation, NHERF1 expression was knocked down in HeLa and CaSki cells, respectively, with the protein levels of NHERF1 reduced up to 90% in both cell lines (Fig. 2a). Depletion of NHERF1 expression significantly enhanced cell proliferation (Fig. 2b) and clonogenicity (Fig. 2c), which was consistent with the results of transfection with two siRNA sequences of NHERF1 individually (Fig. S2). To further verify the results, cell proliferation was detected by CFSE assay. A significant enhancement of proliferation was detected in both HeLa and CaSki cells when NHERF1 expression was depleted compared with the control cells (Fig. 2d). To confirm its inhibitory effects, NHERF1 was ectopically expressed in HeLa and CaSki cells, respectively, and the protein levels of NHERF1 were robustly increased in both cell lines (Fig. 2e). Overexpression of NHERF1 significantly inhibited the clonogenic growth of HeLa and CaSki cells (Fig. 2f), and these data were consistent with the proliferation results from HeLa cells (Fig. S3). Taken together, these findings indicate that NHERF1 inhibits proliferation of cervical cancer cells.

### NHERF1 inhibits cervical cancer cell proliferation through downregulation of ACTN4

**Fig. 3:**
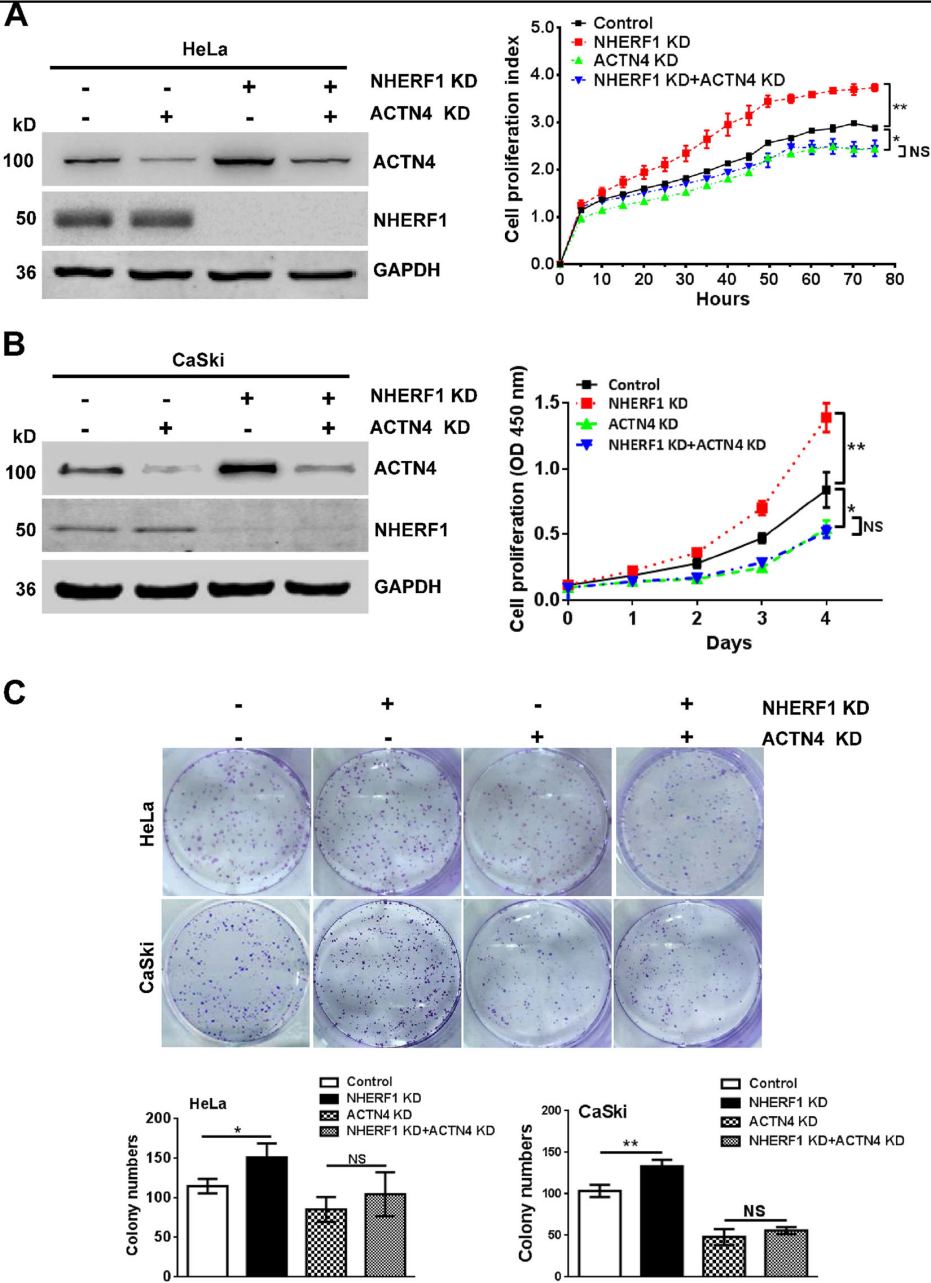
NHERF1 inhibits proliferation of cervical cancer cells through downregulation of ACTN4 expression. **a** Knockdown of NHERF1 promoted HeLa cell proliferation through upregulation of ACTN4. HeLa-NHERF1-KD or HeLa-Control cells were transiently transfected with ACTN4 siRNAs (ACTN4 KD). Cell lysates were analyzed by western blotting with indicated antibodies (left panel). The cell index was determined at the indicated time points by RTCA assay (right panel, repeated-measures analysis of variance, **p *<* 0.01, NS, non-statistical significance, *p* *>* 0.05, error bars represent mean ± s.d., *n* = 3). **b** Knockdown of NHERF1 increased ACTN4 expression level and promoted CaSki cell proliferation. CaSki cells were transfected with ACTN4 siRNAs combined with/without NHERF1 siRNAs. Cell lysates were analyzed by western blotting with indicated antibodies (left penal). The cell proliferations in each group were determined by CCK-8 assay at indicated times points (right panel, repeated-measures analysis of variance, **p *<* 0.01, error bars represent mean ± s.d., *n* = 3). **c** The inhibition of colony formation by NHERF1 was rescued by knockdown of ACTN4 expression in cervical cancer cells. The colony number was monitored in CaSki or HeLa cells after 7 days of culture. Top panel: representative images of cell colonies; bottom panel: quantification of the colony formation efficiency (*t* test; *p *<* 0.05, **p < 0.01, error bars represent mean ± s.d., *n* = 3)

We previously reported that NHERF1 downregulated ACTN4 protein expression levels by promoting ACTN4 ubiquitination and proteasomal degradation^25^. ACTN4 could promote cervical cancer cell proliferation^26^. Thus, it is highly possible that NHERF1 may inhibit proliferation of cervical cancer cells through regulation of ACTN4 protein expression. In order to explore this possibility, the endogenous levels of NHERF1 and ACTN4 in CaSki and HeLa cells were analyzed. We found that CaSki expressed relatively low levels of NHERF1 and high levels of ACTN4 compared with HeLa cells (Fig. S4A), whereas CaSki cells, as expected, exhibited higher proliferation ability than HeLa cells (Fig. S4B–D), implying a potential role of NHERF1 in cervical cancer cell proliferation via regulation of ACTN4. To further verify this hypothesis, proliferation of cervical cancer cells was analyzed after combined depletion of ACTN4 and NHERF1 expression. Data showed that knockdown of NHERF1 expression upregulated ACTN4 protein levels, which were consistent with our previous report^25^, and promoted proliferation of HeLa (Fig. 3a) and CaSki cells (Fig. 3b) as compared with the control. However, when ACTN4 expression was knocked down by siRNA, NHERF1 had less effect on the cervical cancer cell proliferation (Fig. 3a, b and Fig. S5). These results were further confirmed with colony formation assay. Upon depletion of ACTN4 expression, NHERF1 failed to inhibit the clonogenic capacity of cervical cancer cells (Fig. 3c). Taken together, these data indicate that NHERF1 inhibits cervical cancer cell proliferation via suppression of ACTN4 expression.

### NHERF1 suppresses proliferation of cervical cancer cells via inhibition of ACTN4-mediated Wnt/β-catenin signaling activation

**Fig. 4:**
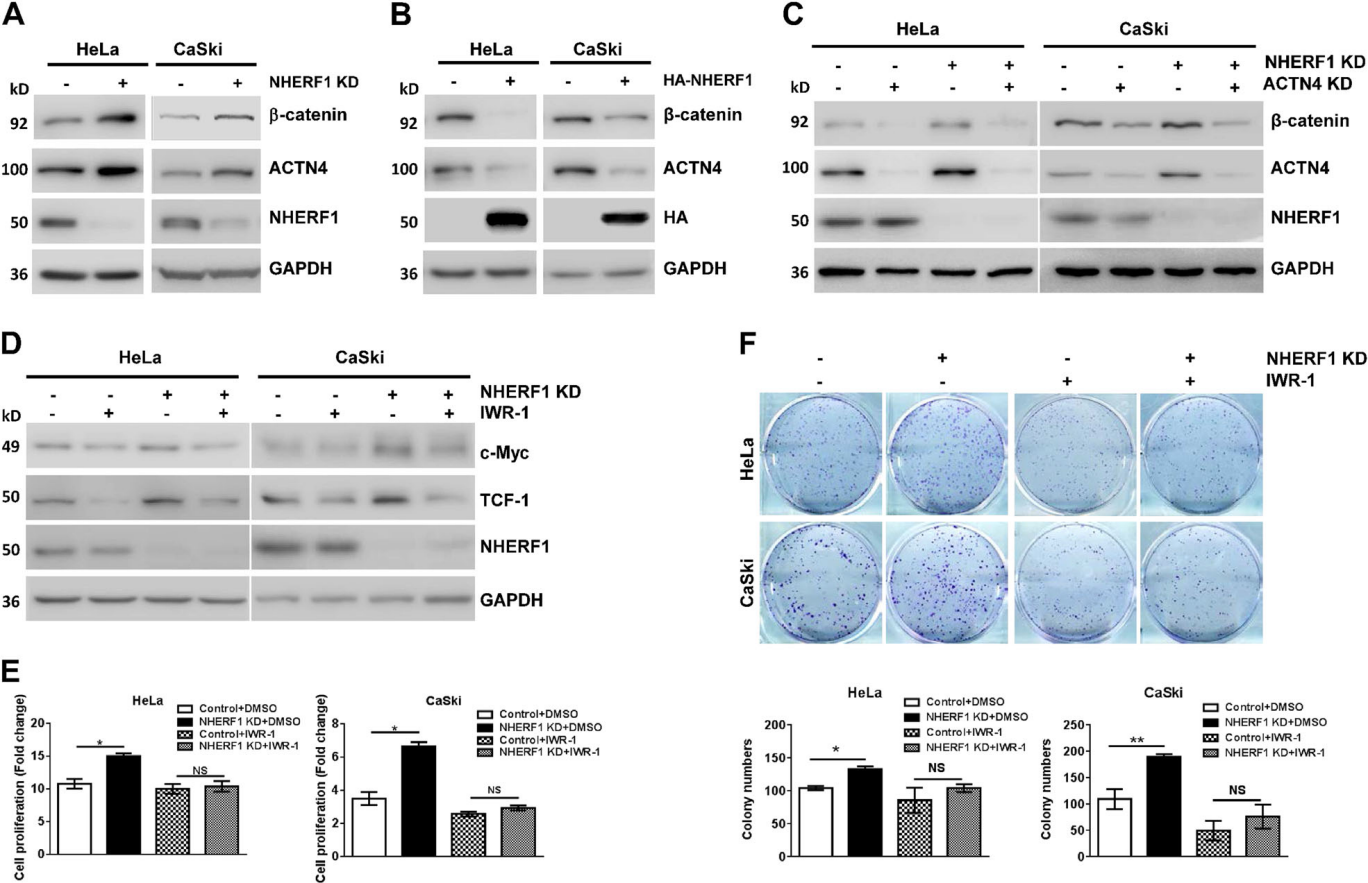
NHERF1 suppresses proliferation of cervical cancer cells through inhibition of Wnt/β-catenin pathway activation via ACTN4. **a** Knockdown of NHERF1 expression increased β-catenin protein level. Cell lysates of HeLa-NHERF1-KD/HeLa-Control or CaSki-NHERF1-KD/CaSki-Control cells were analyzed by western blotting with indicated antibodies. **b** NHERF1 overexpression reduced β-catenin protein level. HeLa and CaSki cells were transiently transfected with NHERF1 constructs, respectively, and the whole cell lysates were immunoblotted with the indicated antibodies. **c** Inhibition of β-catenin expression by NHERF1 required ACTN4 expression. HeLa-NHERF1-KD/HeLa-Control or CaSki-NHERF1-KD/CaSki-Control cells were transfected with siRNAs of ACTN4 or control. Cell lysates were analyzed by western blot with indicated antibodies. **d** Wnt signaling inhibitor abolished NHERF1 inhibition of downstream genes activation of Wnt/β-catenin pathway. HeLa-NHERF1-KD/HeLa-Control or CaSki-NHERF1-KD/CaSki-Control cells were treated with or without IWR-1-endo (IWR-1, 20 μM, 24 h). The cell lysates were subjected to western blot by using specific antibodies. **e** NHERF1 inhibited cervical cancer cell proliferation through Wnt pathway. HeLa-NHERF1-KD/HeLa-Control and CaSki-NHERF1-KD/CaSki-Control cells were treated in the presence or absence of Wnt inhibitor IWR-1 (20 μM) for 72 h, then the proliferation of cells was assessed by CCK-8 assay. Values were represented as relative value after comparing the absorbance at day 3 with that at day 0 (*t* test, *p *<* 0.05, error bars represent mean ± s.d., *n* = 3). **f** NHERF1 inhibited the colony formation of cervical cancer cells via Wnt/β-catenin pathway. The clonogenicity of HeLa-NHERF1-KD/HeLa-Control and CaSki-NHERF1-KD/CaSki-Control cells was analyzed by colony formation assay in the presence or absence of Wnt inhibitor, IWR-1 (20 μM for 7 days). Top panel: typical images of cell colonies; bottom panel: quantification of the colony formation efficiency (*t* test, *p *<* 0.05, **p < 0.01, error bars represent mean ± s.d., *n* = 3)

Recently, Ko *et al*. reported that ACTN4 increased Wnt/β-catenin pathway activation by inhibition of β-catenin degradation, leading to promotion of cervical cancer cell proliferation^26^. NHERF1 was found to associate with Wnt pathway in this study (Fig. 1a). Thus, the regulation of β-catenin protein levels by NHERF1 was further analyzed in cervical cancer cells. Western blot analysis showed that knockdown of NHERF1 indeed increased ACTN4 and β-catenin protein levels in HeLa and CaSki cells (Fig. 4a). Whereas, ACTN4 and β-catenin levels were significantly downregulated when ectopic NHERF1 was expressed (Fig. 4b). To demonstrate whether regulation of β-catenin by NHERF1 required ACTN4, β-catenin levels were analyzed after combined knockdown of NHERF1 and ACTN4. The results showed that silencing NHERF1 expression increased β-catenin levels when ACTN4 existed. However, NHERF1 had less effect on β-catenin levels when ACTN4 was knocked down (Fig. 4c). These data indicated that inhibition of β-catenin expression by NHERF1 required ACTN4. Since β-catenin acts as the central component in canonical Wnt signaling, we further investigated whether NHERF1 could regulate Wnt/β-catenin pathway activation. The expression of c-Myc and TCF-1, downstream target genes of Wnt/β-catenin signaling, was analyzed after treating the cervical cancer cells with IWR-1-endo, a Wnt/β-catenin signaling inhibitor, in the presence or absence of NHERF1 expression. Results showed that knockdown of NHERF1 increased the expression of c-Myc and TCF-1, as we expected. Meanwhile, it failed to regulate the expression of c-Myc and TCF-1 when Wnt inhibitor was applied (Fig. 4d). To illuminate whether NHERF1 inhibited cervical cancer cell proliferation through Wnt pathway, CCK-8 assay was performed by treating the HeLa or CaSki cells with IWR-1-endo combined with/without knockdown of NHERF1 expression. Results showed that depletion of NHERF1 failed to promote cell proliferation when Wnt/β-catenin signaling was blocked by IWR-1-endo in both cells (Fig. 4e). These observations were further verified in colony formation assay. The clonogenic capacity was increased when NHERF1 was depleted. However, clonogenic growth was not affected by NHERF1 when IWR-1-endo was applied (Fig. 4f), indicating that NHERF1 inhibition of cervical cancer cell proliferation was mediated by suppression of Wnt/β-catenin signaling. Taken together, these data suggest that NHERF1 inhibits cervical cancer cells proliferation through reduction of β-catenin levels by regulation of ACTN4 expression.

### NHERF1 inhibits cervical cancer cell proliferation in vivo with the attenuation of Wnt/β-catenin signaling activation

**Fig. 5:**
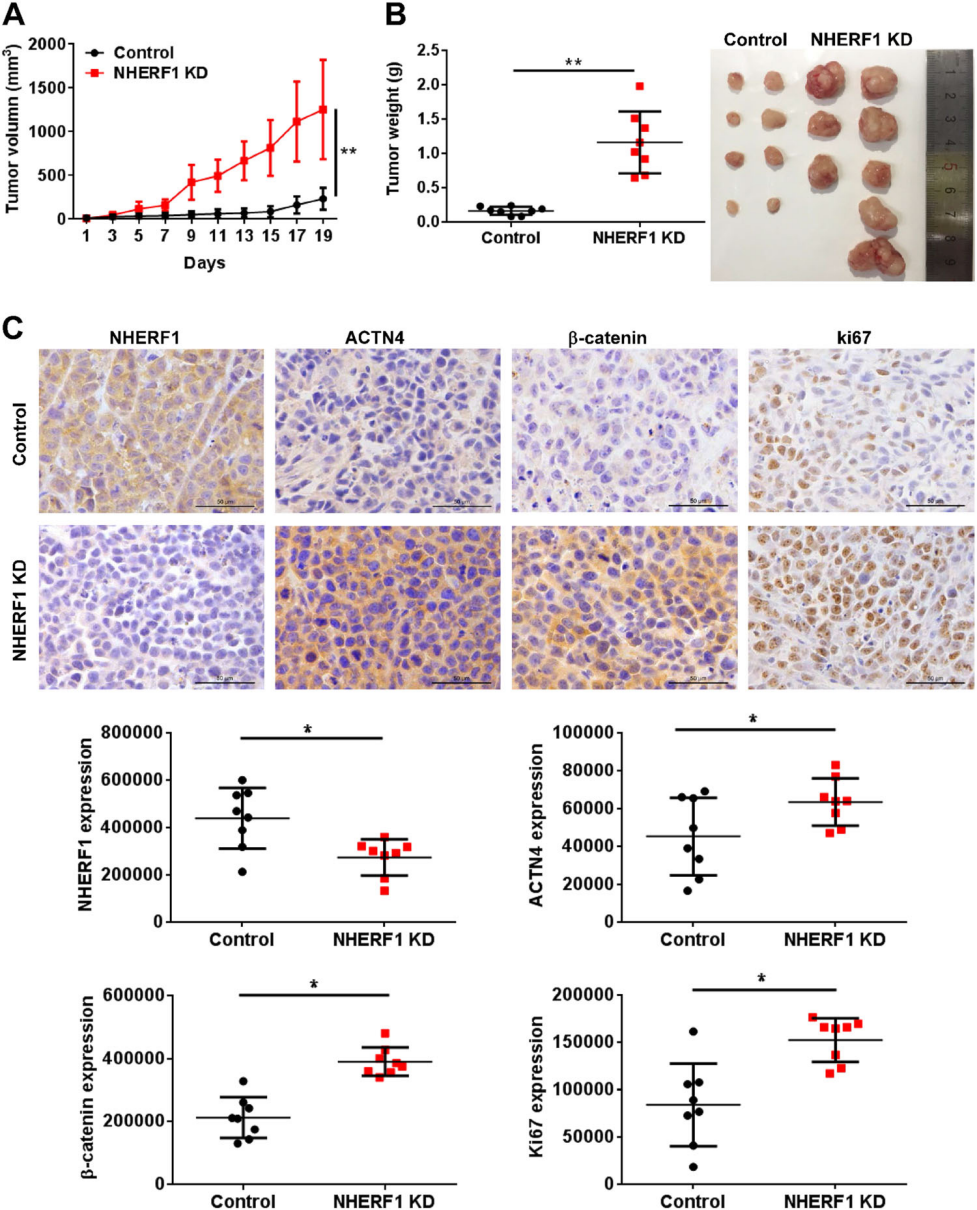
NHERF1 inhibits tumor growth and Wnt/β-catenin pathway activation in xenograft tumors. **a** The growth curve of subcutaneous xenograft tumor from HeLa cells in nude mice. A subcutaneous xenograft tumor model of cervical cancer was established based on HeLa-Control or HeLa-NHERF1-KD cells implantation. Tumor size was measured every 2 days (repeated-measures analysis of variance, **p *<* 0.01, error bars represent mean ± s.d., *n* = 8). **b** Tumor weights of HeLa-NHERF1-KD group were significantly larger than those in the HeLa-Control group. The xenograft tumors were dissected to detect the weights at 20 days after transplantation (left, *t* test, *p *<* 0.05, error bars represent mean ± s.d., *n* = 8), and the image of xenografts was showed on the right. **c** Representative immunohistochemistry staining of NHERF1, ACTN4, β-catenin, and Ki67 of the HeLa-NHERF1-KD group or control xenografts were shown. Scale bar: 50 μm. The quantification of IOD of NHERF1, ACTN4, β-catenin, and Ki67 was obtained by Image-Pro Plus (*t* test, *p *<* 0.05, error bars represent mean ± s.d., *n* = 8)

To examine the roles of NHERF1 in the activation of Wnt/β-catenin signaling and cervical cancer proliferation in vivo, HeLa cells were subcutaneously injected into BALB/c nude mice. Knockdown of NHERF1 robustly enhanced the growth of HeLa xenografts in nude mice within 20 days (Fig. 5). Accordingly, both the volume (Fig. 5a) and weight (Fig. 5b) of tumors were significantly enhanced in NHERF1 knocked down xenografts as compared with the control group. Suppression of NHERF1 in cervical cancer xenograft tumor increased its ACTN4 and β-catenin protein levels, and enhanced the levels of Ki67 by immunohistochemical staining (Fig. 5c). These data suggest that NHERF1 depletion promotes proliferation of cervical cancer xenograft tumor by increasing ACTN4 levels and activation of Wnt/β-catenin pathway.

**Fig. 6:**
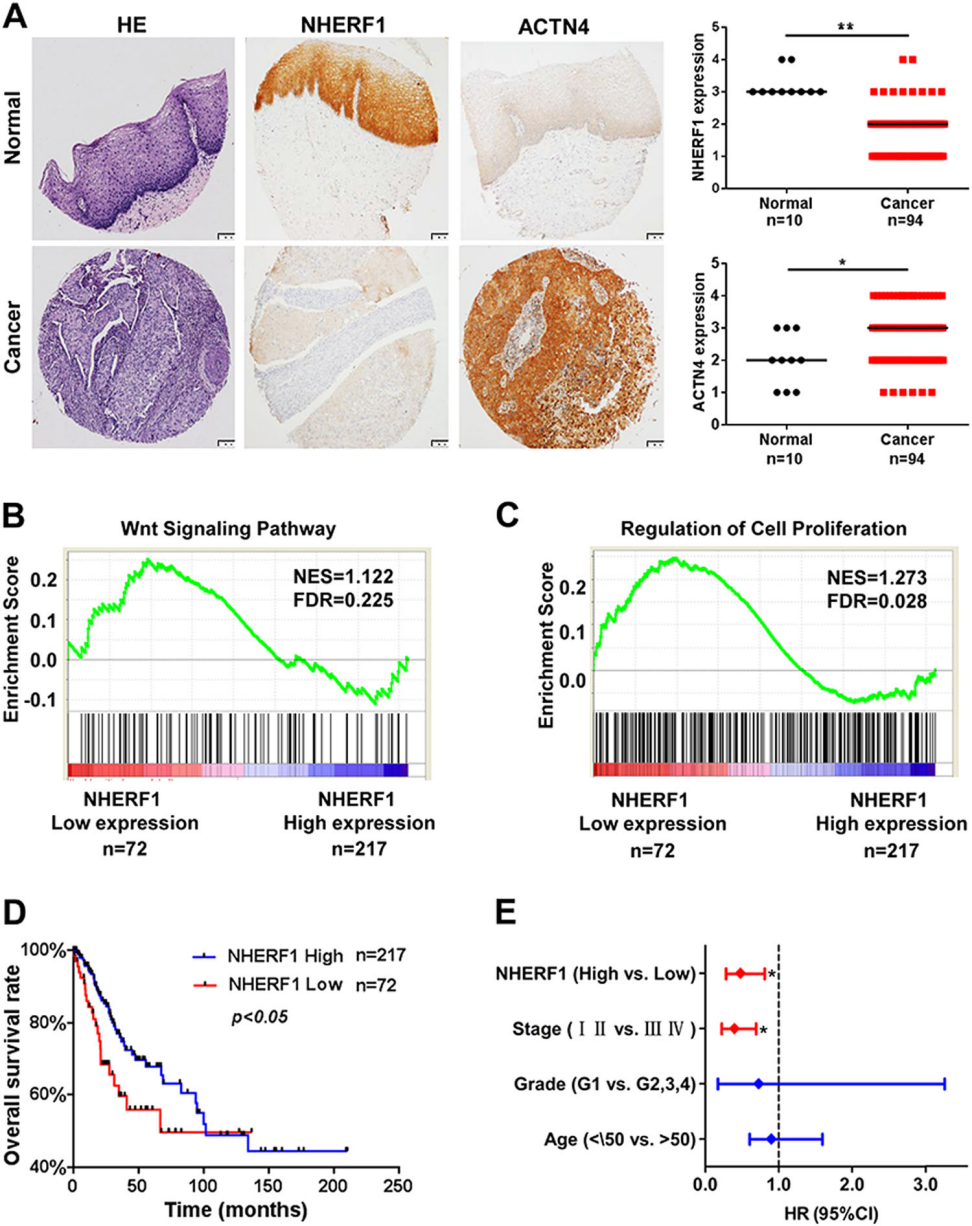
Low levels of NHERF1 expression are associated with Wnt pathway activation, cell proliferation, and poor prognosis of cervical cancer patients. **a** Negative association between the levels of NHERF1 and ACTN4 was detected. The levels of NHERF1 and ACTN4 in cervical cancer and normal cervix tissues were analyzed in tissue microarray CR2083. The left panel was representative images of HE (hematoxylin and eosin) or immunohistochemistry staining of NHERF1 and ACTN4 in tumor and normal cervix tissues. The Scatter plots in the right were analyzed by grading method (nonparametric test, Mann–Whitney test, *p < 0.05, **p < 0.01, error bars represent mean ± s.d.). Scale bar: 100 μm. **b**, **c** NHERF1 expression levels had significant negative correlation with cell proliferation and Wnt signaling activation in cervical cancer specimens. The cervical cancer samples from TCGA database were divided into high and low NHERF1 expression groups according to the lower-quartile value of NHERF1 RNA-seq quantification results. Enrichment plots of gene expression signatures for Wnt pathway activation (**b**) and cell proliferation (**c**) were analyzed by GSEA according to NHERF1 mRNA expression levels. **d** Mantal–Cox analysis of overall survival rates between cervical cancer patients with low or high mRNA levels of NHERF1 in TCGA data set (*p* < 0.05). **e** Low levels of NHERF1 mRNA were an independent risk factor for cervical cancer. NHERF1 expression level, stage, grade, and age were subjected to Cox multivariate regression analysis to identify prognostic factors predictive of overall survival rate. Low levels of NHERF1 and advanced stage (III, IV) were associated with worse survival outcome

To further analyze the association of NHERF1, ACTN4, and Wnt/β-catenin activation in clinical cervical cancer specimens, protein levels of NHERF1 and ACTN4 were examined with immunohistochemical staining. As compared with normal cervix tissues, NHERF1 protein levels were markedly decreased in cervical cancer tissues, which was consistent with results of Fig. 1d, whereas ACTN4 levels were significantly increased (Fig. 6a). Accordingly, the levels of ACTN4, β-catenin, c-Myc, and Ki67 were all increased and NHERF1 levels was deceased in cervical cancer specimens from THPA (www.proteinatlas.org) when compared with normal cervix tissues (Fig. S6). These findings indicate that downregulation of NHERF1 was associated with ACTN4 upregulation and Wnt/β-catenin activation in cervical cancer specimens. To verify the relevance of NHERF1 expression with cell growth and biologic pathways in cervical cancer pathogenesis, GSEA was performed using TCGA cervical cancer data set with the characteristics of patients shown in Supplemental Table I. The cervical cancer patients were stratified by the lower quartile of NHERF1 level in the specimens as high- and low-expression groups. Enrichment plots of GSEA showed that the gene signatures of Wnt/β-catenin signaling activation (Fig. 6b) and cell proliferation (Fig. 6c) were enriched in patients with NHERF1 lower expression. We further evaluated the overall survival of cervical cancer patients in TCGA data via Cox survival analysis. The patients were divided into two groups by NHERF1 levels, and the results showed lower NHERF1 level were correlated with shorter overall survival (Fig. 6d). Cox univariate and multivariate analysis showed that NHERF1 was an independent factor for prognosis prediction of cervical cancer patients (Fig. 6e and Table SII). These findings further indicate that in cervical cancer specimens, NHERF1 expression is negatively associated with Wnt/β-catenin activation and cell proliferation.

## Discussion

Hyperactivation of Wnt/β-catenin signaling pathway has been implicated in the cancerous proliferation of various types of cancer including cervical cancer^10,12^. In the present study, *NHERF1* was a novel downregulated gene involved in Wnt signaling and cell proliferation in cervical cancer (Fig. 1). The molecular mechanisms of NHERF1 in the regulation of Wnt/β-catenin signaling and the proliferation of cervical cancer were largely unknown.

NHERF1 is extensively expressed in the epithelium of tissues and has been found to be implicated in various types of cancer. Extensive studies suggest an oncogenic role of NHERF1 in breast cancer^27–29^, ovarian mucinous carcinoma^30^, hepatocellular carcinoma^31^, and glioblastoma^32^. On the contrary, several reports show that NHERF1 acts as a tumor suppressor in esophageal squamous cell carcinoma^33^ and triple-negative breast cancer^34^. In the present study, we found that NHERF1 inhibited cervical cancer cell proliferation from in vitro (Fig. 2 and Fig. S2,3) and in vivo models (Fig. 5), suggesting an anti-proliferation and tumor-suppressive effect of NHERF1 in cervical cancer. NHERF1 has emerged as a key regulator of cancer signaling network via assembling cancer-associated proteins^35^. In previous study, we demonstrated that NHERF1 interacted with ACTN4 and downregulated the expression levels of ACTN4^25^. Study from other groups showed that upregulation of ACTN4 enhanced proliferation of cervical cancer cells in cellullar and xenograft models by promoting stability of β-catenin through phosphorylation of Akt and GSK3β^26,36^. In the present study, we further revealed that NHERF1 inhibition of cervical cancer cell proliferation was mediated via ACTN4 (Fig. 3 and Fig. S5). These findings have provided further insights into the role of ACTN4 in cancer cell proliferation apart from its roles in maintaining cytoskeletal integrity^37^.

Activation of Wnt/β-catenin signaling pathway is significantly associated with the cell proliferation and poor prognosis of cervical cancer^17,21^. The present in vitro data showed that NHERF1 downregulated the levels of β-catenin by suppression of ACTN4 expression (Fig. 4a–c). Blocking Wnt/β-catenin signaling abolished the enhancement of cervical cancer cell proliferation induced by knockdown of NHERF1 (Fig. 4d–h). Data from in vivo mouse models and clinical specimens showed prominent downregulation of NHERF1 and upregulation of ACTN4, β-catenin, and its downstream targets (Figs. 1, 5, 6a and Fig. S6). Further analysis revealed that lower levels of NHERF1 were prominently correlated with activation of Wnt/β-catenin signaling and cell proliferation (Fig. 6b, c), and were an independent risk factor for worse prognosis of cervical cancer patients (Fig. 6d, e). NHERF1 loss has also been reported to associate with the activation of other oncogenic pathways, such as the ERK^38^ and Akt signaling^39^ in cervical cancer cells. However, there was no association between ERK or Akt signaling activation and the overall survival of cervical cancer patients in TCGA database (data not shown). All these findings suggest that NHERF1 may suppress Wnt/β-catenin signaling activation via a decrease in ACTN4 levels to elicit anti-proliferation and tumor-suppressive effects in cervical cancer. It is likely that downregulation of NHERF1 may result in development of cervical cancer by promotion of β-catenin-mediated proliferation. Therefore, NHERF1 may potentially serve as a biomarker for prognosis evaluation or a therapeutic target of cervical cancer.

Cisplatin-based chemotherapy is the standard treatment for the advanced stage and recurrent cervical cancer^1^. However, chemoresistance seriously compromises the efficacy of cisplatin^40^. Therefore, cisplatin resistance has become a major clinical challenge. Recently, increasing evidences indicate that overactivation of Wnt signaling pathway has been implicated in resistance to chemotherapy^41,42^. In the present study, results showed that cisplatin resistance was associated with dysregulation of Wnt signaling in HeLa cells (Fig. S7A), which further indicated that Wnt signaling may play a key role in cisplatin resistance in cervical cancer. However, the detailed mechanisms of Wnt signaling in cisplatin resistance are still far from clear. In this study, we showed that both gene signatures of cisplatin resistance and Wnt signaling were enriched in NHERF1 low-expression cervical cancer patients (Fig. S7B,C). Further results showed that activation of downstream genes of cisplatin resistance and Wnt signaling was more profound in cisplatin-resistant patients (Fig. S7D,E). We previously reported that low levels of NHERF1 expression were associated with cisplatin resistance in cervical cancer^39^. Taken together, we proposed a possible molecular mechanism for cisplatin resistance in cervical cancer by which downregulation of NHERF1 promotes overactivation of Wnt/β-catenin signaling and results in cisplatin resistance. Further study is needed to confirm this hypothesis.

In the present study, significant worse prognosis was found in cervical cancer patients with lower levels of NHERF1 (Fig. 6d). It was surprising to find that proportion of HPV-inactive patients was significantly higher than HPV-active group in NHERF1 low-expression cervical cancer patients (Fig. S8A and Table SIII). HPV-inactive cervical cancer patients had worse prognosis than HPV-active group (Fig. S8B and Table SIV), in accordance with findings from Banister* et al*. that activation of Wnt/β-catenin signaling was associated with worse prognosis of HPV-inactive cervical cancer patients^16^. However, the molecular mechanisms underlying the development of HPV-inactive cervical carcinoma are still elusive. Our data further showed that the mRNA levels of NHERF1 in HPV-inactive cervical cancer patients were significantly lower and activation of Wnt/β-catenin signaling and proliferation genes were more prominent in this subgroup of patients (Fig. S8C–E). Therefore, it is likely that the worse prognosis of HPV-inactive cervical cancer patients may be attributed to robustly low levels of NHERF1. These findings suggest that overactivation of Wnt/β-catenin signaling in response to the significant downregulation of NHERF1 at mRNA levels may contribute to the development and progression of HPV-inactive cervical cancer.

In HPV-active cervical cancer, activation of Wnt/β-catenin signaling and cellular proliferation was more remarkable in patients with poor prognosis (Fig. S9B,C). However, there was no difference of the mRNA levels of NHERF1 between patients with good or poor prognosis (Fig. S9A). This seems in contradiction with the results from present study. Several studies reported that oncogenic E6 and E7 proteins of HR-HPV could downregulate the levels of target proteins via regulation of the target stability^43,44^. For example, HPV16-E6 protein has been found to degrade NHERF1 protein at posttranslational level^45^. Therefore, it is reasonable to speculate that higher the oncogenic activities of HR-HPV-E6, the more powerful degradation of NHERF1 protein. Downregulation of NHERF1 protein then leads to oncogenenic proliferation upon upregulation of ACTN4 and activation of Wnt/β-catenin signaling in HPV-active cervical cancer. This possibility needs to be verified by comparison of the mRNA and protein levels of NHERF1, activation status of Wnt/β-catenin signaling, and prognosis of HPV-active cervical cancer in the future study.

In summary, the present study provides novel evidences for a tumor-suppressive role of NHERF1 in cervical cancer cell proliferation by attenuation of Wnt/β-catenin signaling via a decrease in ACTN4 expression. Low level of NHERF1 may contribute to the development of cervical cancer and indicated poor prognosis of cervical cancer patients. These findings could also improve our understanding for the molecular mechanisms of cisplatin resistance, and the development and prognosis of HPV-inactive and HPV-active cervical cancer.

## Materials and methods

### Cell culture and transfection

Cells were purchased from the National Infrastructure of Cell Line Resource (Beijing, China). HeLa and CaSki cervical cancer cells were cultured in DMEM, RPMI 1640 medium (Gibico, Cleveland, TN), respectively, with 10% FBS (Gibico, Cleveland, TN) at 37 ℃ in an atmosphere of 5% CO_2_. The stable transfection of HeLa cells was performed as previously described^25^.

### RNA interference and plasmid constructions

siRNAs were purchased from Invitrogen (Carlsbad, CA) and the sequences were shown as follows:

NHERF1 siRNA1#: 5′-GCUAUGGCUUCAACCUGCATT-3′.

NHERF1 siRNA2#: 5′-GUCGACCACCAGCAGGCGCACGGCGUUG-3′.

ACTN4 siRNA1#: 5′-CCUGAACAAUGCCUUCGAATT-3′.

ACTN4 siRNA2#: 5′-CCUGAACAAUGCCUUCGAATT-3′.

Negative control siRNA1#: 5′-UUCUUCGAACGUGUCACG-3′.

Negative control siRNA2#: 5′-UCCAGACGGCGCAGUGGGCGACCGCUAC-3′.

Cells were transfected with a mixture of two siRNA sequences for relevant RNA interference experiments. NHERF1 was stably knocked down by shNHERF1 in HeLa (Hela-NHERF1-KD) cells and transiently knocked down by siRNA in CaSki (CaSki-NHERF1-KD) cells.

pCMV-HA and pCMV-HA-NHERF1 plasmids were kindly provided by Dr. Randy Hall (EmoryUniversity, Atlanta, GA). pSuper.puro luciferase control and pSuper.puro-shNHERF1 plasmids were kind gifts of Dr. Margaret J. Wheelock (University of Nebraska Medical Center, Omaha, NE).

### Western blotting and reagents

Western blotting assay was performed as previously described^46^. The anti-NHERF1 was purchased from Sigma-Aldrich (HPA009672, St. Louis, MO) and Becton Dickinson Labware (#611161, Billerica, MA), respectively. Anti-HA (#561) was purchased from Medical & Biological Laboratories (Nagoya, Japan). Anti-c-Myc (#ab32072) and anti-β-catenin (#ab22656) were purchased from Abcam (Cambridge, UK). Anti-GAPDH (#5174), anti-β-catenin (#9581), and anti-TCF-1 (#2206) were purchased from Cell Signaling Technology (Danvers, MA). Anti-ACTN4 was purchased from Enzo Life Sciences (#ALX-210-356, Shanghai, China) and Santa Cruz Biotechnology (#sc-134236, Santa Cruz, CA), respectively. Anti-Ki67 (#zm-0166) and horse radish peroxidase-conjugated secondary antibodies were purchased from ZSGB-BIO (Beijing, China). Infrared fluorescent dyes-conjugated secondary antibodies were purchased from LI-COR Biosciences (Lincoln, NE). IWR-1-endo (#S7086) was purchased from Selleck (Houston, TX).

### Cell proliferation assay

For CCK-8 assay, cells were seeded in 96-well plates at a density of 3000 per well and cultured for 1–5 days, and CCK-8 (Dojindo, Kumamoto, Japan) was added according to the manufacturer’s instructions and absorbance was measured at 450 nm with an EnSpire label microplate reader (PerkinElmer, Waltham, MA).

For CFSE (carboxy fluorescein succinimidyl ester) assay, single-cell suspension at a density of 1 × 10^6^ cells per ml was stained with CFSE Cell Proliferation Kit (#C34554, Life Technologies, Carlsbad, CA) at day 1 and continued the culture for 3 days. The labeling cells were analyzed by flow cytometry at day 1 and day 3, respectively. The proliferation index of each group was analyzed by comparing the fluorescence value of day 1 and day 3 by Modfit LT (Verity Software House, Topsham, ME).

For RTCA (real-time cell analysis) assay, cells were cultured in 16-well plates (3000 cells per well, E-Plate 16, ACEA Biosciences Inc) and proliferation index was monitored by the xCELLigence system (ACEA Biosciences Inc, San Diego, CA) for 72 h.

For colony formation assay, cells were cultured in 6-well plates (1000 cells per well) for 7 days. The number of colonies (> 50 cells) were counted after staining with 0.5% crystal violet.

### In vivo xenograft formation assay

This study was performed following the Guide for the Care and Use of Laboratory Animals by National Institutes of Health, and all procedures were approved by the Animal Care and Use Committee of Capital Medical University.

BALB/c nude mice (5 weeks, female) were subcutaneously injected with HeLa-NHERF1-KD or control cells (1 × 10^5^ cells in 0.1 mL PBS per mouse, 8 mice per group) and monitored every 2 days for the growth of tumors (volume = (length × width^2^)/2). Mice were killed 20 days after the injection. The tumor xenografts were embedded in paraffin for further studies.

### Immunohistochemical staining

The tissue microarrays were immunostained with anti-NHERF1 (HPA009672) and anti-ACTN4 (sc-134236), respectively. The intensity of immunostaining in individual tumor tissue (0, no staining; 1, weak staining; 2, moderate staining; 3, strong staining), and the staining percentage of tumor tissue (0, none; 1, 1%–25%; 2, 26%–50%; 3, 51%–75%; 4, >75%) were scored. The absolute value of the protein expression levels was thus classified into four grades after multiplying the corresponding intensity value with percentage scores: I (grades 0–3), II (grades 4–6), III (grades 7–9), and IV (grades 10–12).

Xenograft tumor tissues were stained by anti-NHERF1, anti-ACTN4, anti-β-catenin (#ab22656), or anti-Ki67 antibody, respectively. The average IOD (integrated optical density) of five fields of each sample was analyzed by Image-Pro Plus (Rockville, MD) and represented the expression level of protein.

### Data set collection and cervical cancer patient samples

The tissue microarrays (CR2083, containing biopsies from 94 cases of cervical cancer and 10 normal cervical tissues) were purchased from Biomax, Xi’an Alenabio (Xi’an, Shanxi, China), and the tissue microarrays (OD-CT-RpUtr03-006, containing cervical cancer and adjacent normal tissues biopsies from 31 stage III cervical cancer patients) were purchased from Shanghai outdo biotech co., LTD. GSE26342 and GSE9750 from GEO (two of the largest cervical cancer data sets with more than 20 cases of normal cervix tissues and 30 cases of cervical cancer patients) were used for the screening of differential expression genes by significance analysis of microarrays (http://statweb.stanford.edu/~tibs/SAM/) and DAVID analysis (https://david.ncifcrf.gov). GSE151120 was used for GO (gene ontology) analysis by protein analysis through evolutionary relationships (http://pantherdb.org/webservices/go/overrep.jsp). GSE89657 and GSE9750 from GEO were used to analyze the NHERF1 mRNA expression level in cervical cancer cell lines. RNA-Seq data from TCGA cervical cancer patients were collected from Synapse website (http://synapse.org;syn1571569). Clinical data were downloaded from cBioPortal database (http://www.cbioportal.org). Data of HPV-infected status were obtained from the study by Banister et al. HPV-active: with high levels of E6/E7 expression; HPV-inactive: with low or zero E6/E7 expression^16^. All the samples obtained from GSE26342, GSE9750, and TCGA data sets were untreated primary cervical cancer.

The immunohistochemistry-based protein expression images of cervical cancer and normal cervix tissues were downloaded from THPA (The Human Protein Atlas) (www.proteinatlas.org) and analyzed by grading method.

### Gene set enrichment analysis (GSEA)

GSEA (www.broad.mit.edu/gsea) protocol was performed as previously described^47^. The association between phenotypes, biological processes/pathway, and protein mRNA expression level were analyzed. Pre-defined gene set were obtained from the Molecular Signatures Database, MSigDB (http://software.broadinstitute.org/gsea/msigdb). Gene sets: GO_CANONICAL_WNT_SIGNALING_PATHWAY (M12752), GO_REGULATION_OF_EPITHELIAL_CELL_PROLIFERATION (M12114), KANG_CISPLATIN_RESISTANCE_UP (M2767). The FDR (false discovery rate) score smaller than 0.25 was considered significant enrichment of a gene set^48^.

### Statistical analysis

All statistical analyses were carried out using the GraphPad prism 5 (Graphpad Software, Inc., La Jolla, CA) or SPSS (SPSS Inc, Chicago, IL). It was considered as statistical significant when *p* *<* 0.05 (*p* *<* 0.05 and *p* *<* 0.01 are designated by * and **, respectively).

## Electronic supplementary material

supplemental information
