# Supplementary material for: NHERF1 inhibits beta-catenin-mediated proliferation of cervical cancer cells through suppression of alpha-actinin-4 expression
Source: Cell Death Dis. 2018 Jun 4;9(6):668. doi: 10.1038/s41419-018-0711-x (PMC5986762; doi:10.1038/s41419-018-0711-x)
Supplement: Supplementary file 1 — supplemental information [file 41419_2018_711_MOESM1_ESM.pdf]

Supplemental figure legends

Figure S1. The mRNA levels of NHERF1 in multiple cervical cancer cell lines. (A-B) Relative NHERF1 mRNA levels of cervical cancer cell lines from GSE9750 (A) and GSE89657 (B). (C) The protein levels of NHERF1 in HeLa and CaSki cells were detected by Western blot with indicated antibodies.

Figure S2. Knock down of NHERF1 enhances proliferation of HeLa cells. (A) Knock down of NHERF1 expression in cervical cancer cells was verified by immunoblotting analysis. HeLa cells were transiently transfected with NHERF1 siRNA#1 and #2 respectively. Cell lysates were immunoblotted with the indicated antibodies in the figure. (B) Knock down of NHERF1 enhanced proliferation of HeLa cells. HeLa cells were subjected to the CCK-8 assay. Values represent the absorbance at 450 nm (repeated-measures analysis of variance, ** $p < 0.01$, error bars represent mean ± s.d., n=3) (C) Knock down of NHERF1 enhanced the colony formation of HeLa cells. Colony formation was monitored in HeLa cells for 7 days. Left panel: Representative photographs of the colonogenicity. Right panel: Quantification of the colony formation efficiency (*t* test, * $p < 0.05$, error bars represent mean ± s.d., n=3).

Figure S3. NHERF1 overexpression inhibits proliferation of HeLa cells. HeLa cells were transiently transfection with NHERF1 constructs and cell proliferation was detected by CCK-8 assay at indicated time points (repeated-measures analysis of variance, ** $p < 0.01$, error bars represent mean ± s.d., n=3).

Figure S4. CaSki exhibits higher proliferation ability, with relatively low levels of NHERF1 and high levels of ACTN4 as compared with HeLa cells. (A) Endogenous expression of NHERF1 and ACTN4 in cervical cancer cells was verified by Western blot analysis. Proliferation ability of cervical cancer cells was analyzed by assays of CCK8 (B) (repeated-measures analysis of variance, ** $p < 0.01$, error bars represent mean ± s.d., n=3), colony formation (C) (*t* test, ** $p < 0.01$, error bars represent mean ± s.d., n=3) and RTCA (5000 cells per well, repeated-measures

analysis of variance, ** $p < 0.01$, error bars represent mean ± s.d., n=3) (D) respectively.

Figure S5. Downregulation of NHERF1 promoted CaSki cells proliferation through regulation of ACTN4 expression. CaSki cells were transfected with ACTN4 siRNAs combined with/without NHERF1 siRNAs. The cell proliferation in each group was determined by RTCA assay at indicated time points (repeated-measures analysis of variance, ** $p < 0.01$, error bars represent mean ± s.d., n=3).

Figure S6. Upregulation of ACTN4, β-catenin and c-Myc is verified in cervical cancer tissues. (A) Protein levels of NHERF1, ACTN4, β-catenin, c-Myc and Ki67 in human cervical cancer specimens and normal cervical tissues were obtained from THPA. The scatter plots of the above proteins were quantified by grading method described in method (nonparametric test, Mann-whitney test, * $p < 0.05$, ** $p < 0.01$, error bars represent mean ± s.d.).

Figure S7. Activation of cell proliferation and Wnt signaling genes is significantly correlated with cisplatin-resistance. (A) Functional clustering analysis revealed a significant enrichment of Wnt signaling pathway genes in cervical cancer cisplatin resistance in GSE15120 by Protein Analysis through Evolutionary Relationships analysis. (B-C) Low levels of NHERF1 expression were associated with cisplatin resistance and Wnt pathway activation. Cervical cancer patients with cisplatin treatment of TCGA database were divided into high and low NHERF1 expression groups according to the median value of NHERF1 RNA-seq quantification results. Enrichment plots of gene signatures for cisplatin resistance (KANG_CISPLATIN_RESISTANCE_UP, M2767) (B) and Wnt pathway activation (C) according to the NHERF1 mRNA levels by GSEA of TCGA cervical cancer database. (D-E) Activation of cisplatin resistance and Wnt pathway was correlated with poorer prognosis of cervical cancer patients with cisplatin treatment. Cervical

cancer patients with cisplatin treatment were divided into cisplatin resistant and sensitive groups. GSEA plot for gene expression signatures for cisplatin resistance (D) and Wnt pathway (E) according to the disease free survival of patients with cisplatin treatment in TCGA cervical cancer data set. Resistant: Recurred/Progress; Sensitive: Disease Free > 3years.

Figure S8. Lower levels of NHERF1 are detected with higher frequency in HPV-inactive cervical cancer patients as compared with HPV-active group. (A) The proportion of HPV-inactive cases in NHERF1 low expression cervical cancer patients was higher compared with NHERF1 high expression group by Fisher's exact analysis. (B) HPV-inactive is an independent risk factor for cervical cancer. HPV status, stage, grade, and age were subjected to Cox multivariate regression analysis. HPV inactive and advanced stages were significantly associated with poorer overall survival rate of cervical cancer patients. (C) Downregulation of NHERF1 is more prominent in HPV-inactive specimens as compared with HPV-active group in TCGA cervical cancer data set (nonparametric test, Mann-whitney test, **$p < 0.01$). (D-E) NHERF1 expression levels had significant negative correlation with Wnt signaling and cell proliferation activation in HPV-inactive cervical cancer specimens. Enrichment plots of gene expression signatures for Wnt pathway (D, GO_REGULATION_OF_WNT_SIGNALING_PATHWAY, M11440) and cell proliferation (E) according to NHERF1 mRNA levels in HPV-inactive cervical cancer patients by GSEA of TCGA database. The median level of NHERF1 mRNA was used as a cut-off value.

Figure S9. Aberrant activation of Wnt/β-catenin signaling and cell proliferation is correlated with poorer prognosis of HPV-active cervical cancer patients. (A) The mRNA levels of NHERF1 were not associated with the prognosis of HPV-active cervical cancer patients. The mRNA levels of NHERF1 were compared between patients with poor or good prognosis in HPV-active cervical cancer (living > 3years: good prognosis; deceased < 2 years: poor prognosis, nonparametric test,

Mann-whitney test, NS: non statistical significance, $p > 0.05$). (B-C) Activation of Wnt signaling and cell proliferation genes are positively correlated with poor prognosis of HPV-active cervical cancer patients. Enrichment plots of gene expression signatures for Wnt/β-catenin signaling (B) and cellular proliferation (C) were analyzed by GSEA in TCGA cervical cancer database

Table S Ⅰ Clinical information of cervical cancer patients

	cervical cancer patients
Characteristic	
Patient, no.	306
Age, years, mean (range)	48 (20-88)
Pathological stage，no. (%)	
I	162 (52.9)
II	70 (22.9)
III	46 (15.0)
IV	21 (6.9)
Unknown	7 (2.3)
Pathological grade, no. (%)	
G1	18 (5.9)
G2	136 (44.4)
G3	119 (38.9)
G4	1 (0.3)
GX	24 (7.8)
Unknown	8 (2.6)
Patient with all clinical data, no. (%)	289 (94.4)
HPV no. (%)	
Inactive	19 (6.2)
Active	227 (74.2)
Unknown	60 (19.6)

Table SⅡ Univariate and multivariate analysis of NHERF1 level and patient OS

	Univariate analysis			Multivariate analysis[c]		
Variable	HR[a]	95% CI[b]	*p*	HR	95% CI	*p*
Overall survival (n = 289)						
Age (years)						
≤50 (n=178)	0.734	0.460-1.171	0.194	0.899	0.551-1.466	0.669
>50 (n=111)						
Grade						
G1(n=18)	0.509	0.125-2.082	0.348	0.728	0.175-3.027	0.663
G2, G3, G4, GX (n=271)						
Stage						
Ⅰ, Ⅱ (n=225)	0.442	0.270-0.724	0.001	0.397	0.233-0.679	0.001
Ⅲ, Ⅳ (n=64)						
NHERF1						
High (n=217)	0.573	0.347-0.945	0.029	0.479	0.284-0.808	0.006
Low (n=72)						

a Hazard ratio, estimated from Cox proportional hazard regression model.
b Confidence interval of the estimated HR.
c Multivariate models were adjusted for Stage classification, Grade classification, and age.

Table SⅢ HPV status of cervical cancer patients in NHERF1 low or high expression groups

Variables	HPV inactive (%)**	HPV active (%)	HPV unknown (%)	Patients (n)
NHERF1 Low	14 (19.4)	48 (66.7)	10 (13.9)	72
NHERF1 High	5 (2.3)	179 (82.5)	33 (15.2)	217

** HPV inactive *vs.* HPV active, Fisher's exact test, $p < 0.001$

Table SⅣ Univariate and multivariate analysis of HPV status and patient OS

	Univariate analysis			Multivariate analysis[c]		
Variable	HR[a]	95% CI[b]	*p*	HR	95% CI	*p*
Overall survival (n = 246)						
Age (years)						
≤50 (n=150)	0.803	0.464-1.389	0.432	1.094	0.616-1.942	0.76
>50 (n=96)						
Grade						
G1 (n=16)	0.281	0.039-2.036	0.209	0.373	0.051-2.735	0.332
G2, G3, G4, GX (n=230)						
Stage						
Ⅰ, Ⅱ (n=190)	0.390	0.222-0.684	0.001	0.369	0.204-0.665	0.001
Ⅲ, Ⅳ (n=56)						
HPV						
Active (n=227)	0.477	0.214-1.061	0.069	0.412	0.181-0.934	0.034
Inactive (n=19)						

a Hazard ratio, estimated from Cox proportional hazard regression model.

b Confidence interval of the estimated HR.

c Multivariate models were adjusted for Stage classification, Grade classification, and age.

Figure S1

A

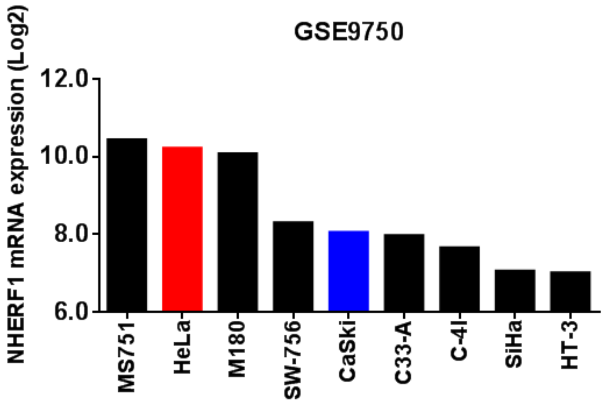

B

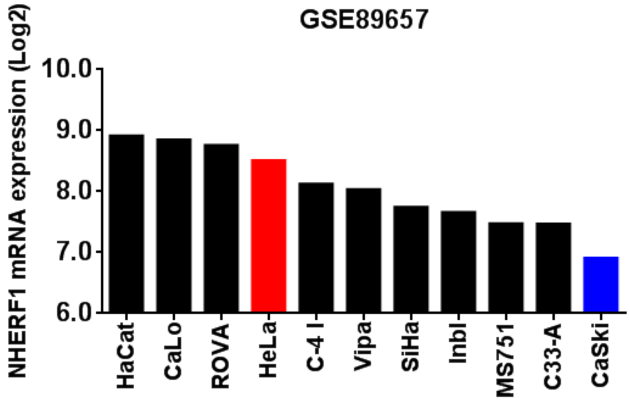

C

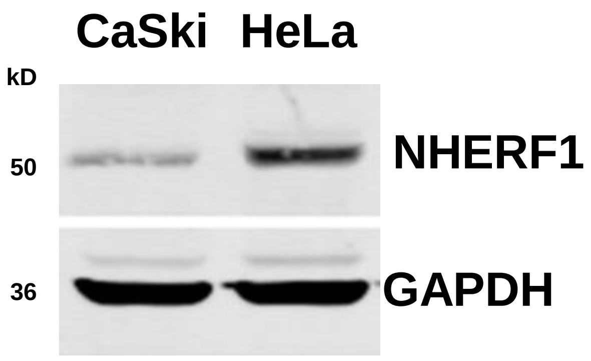

Figure S2

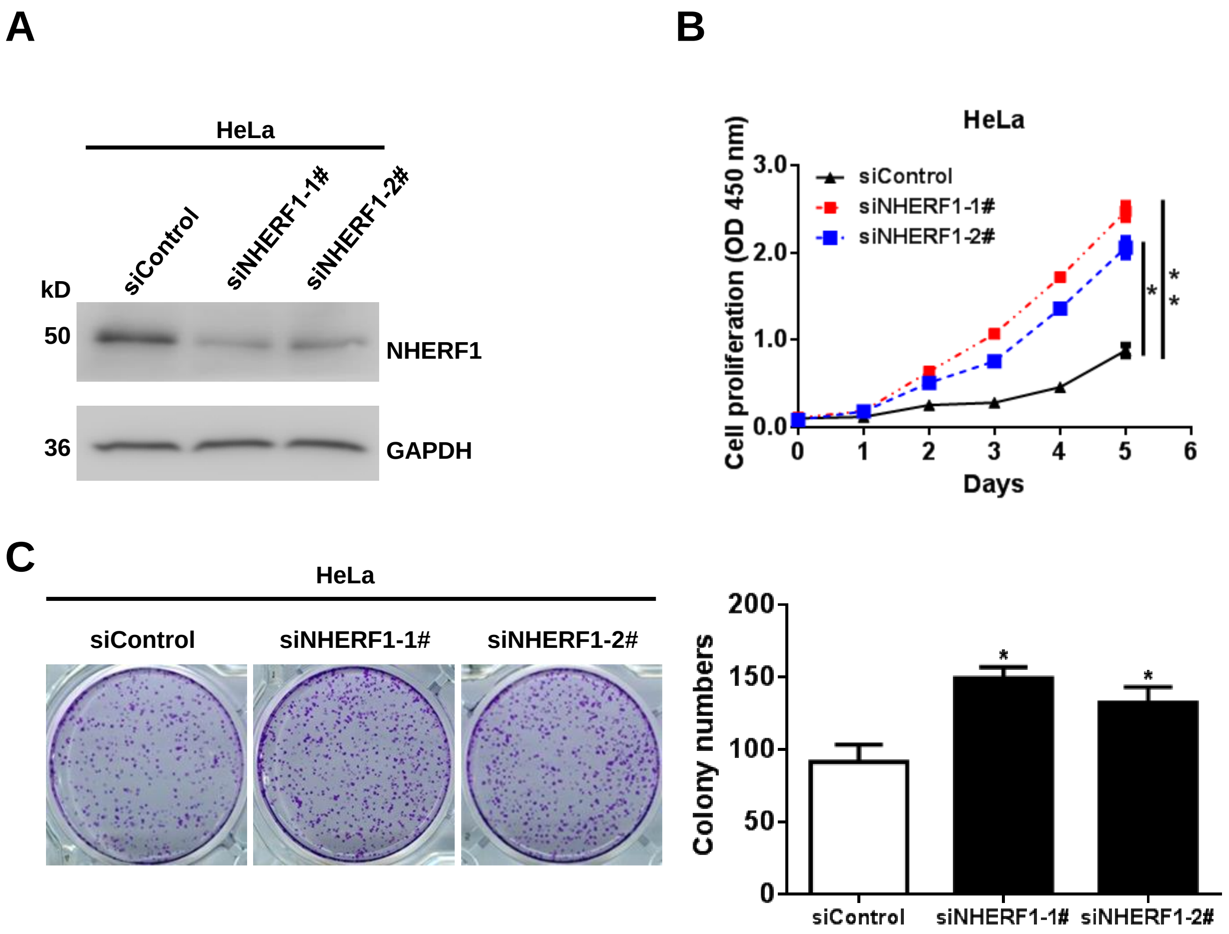

Figure S3

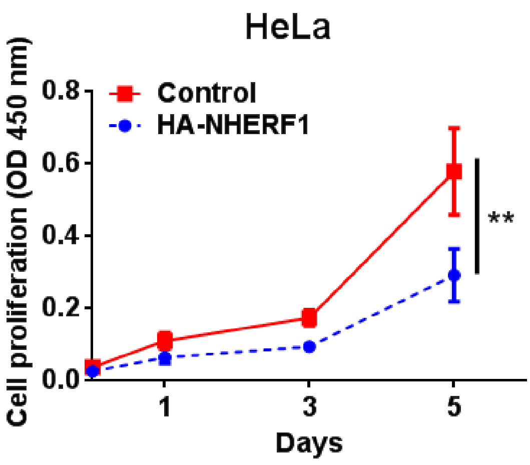

Figure S4

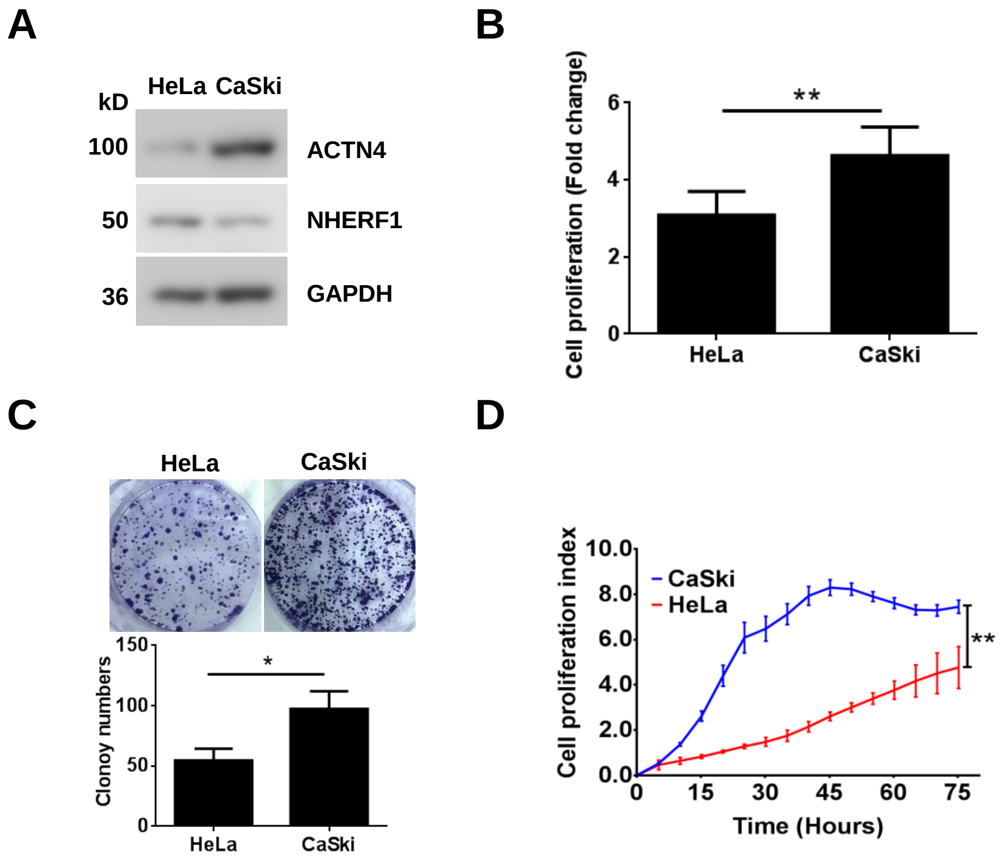

Figure S5

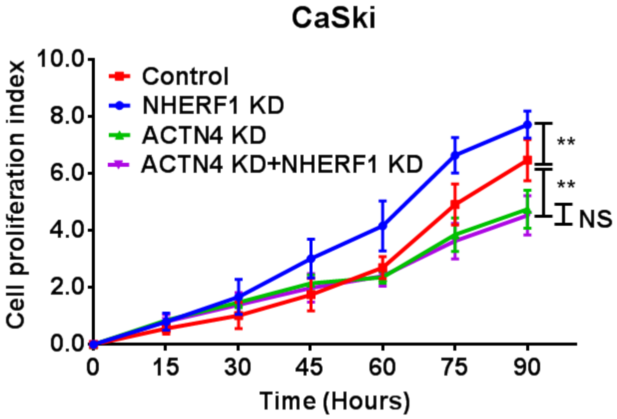

Figure S6

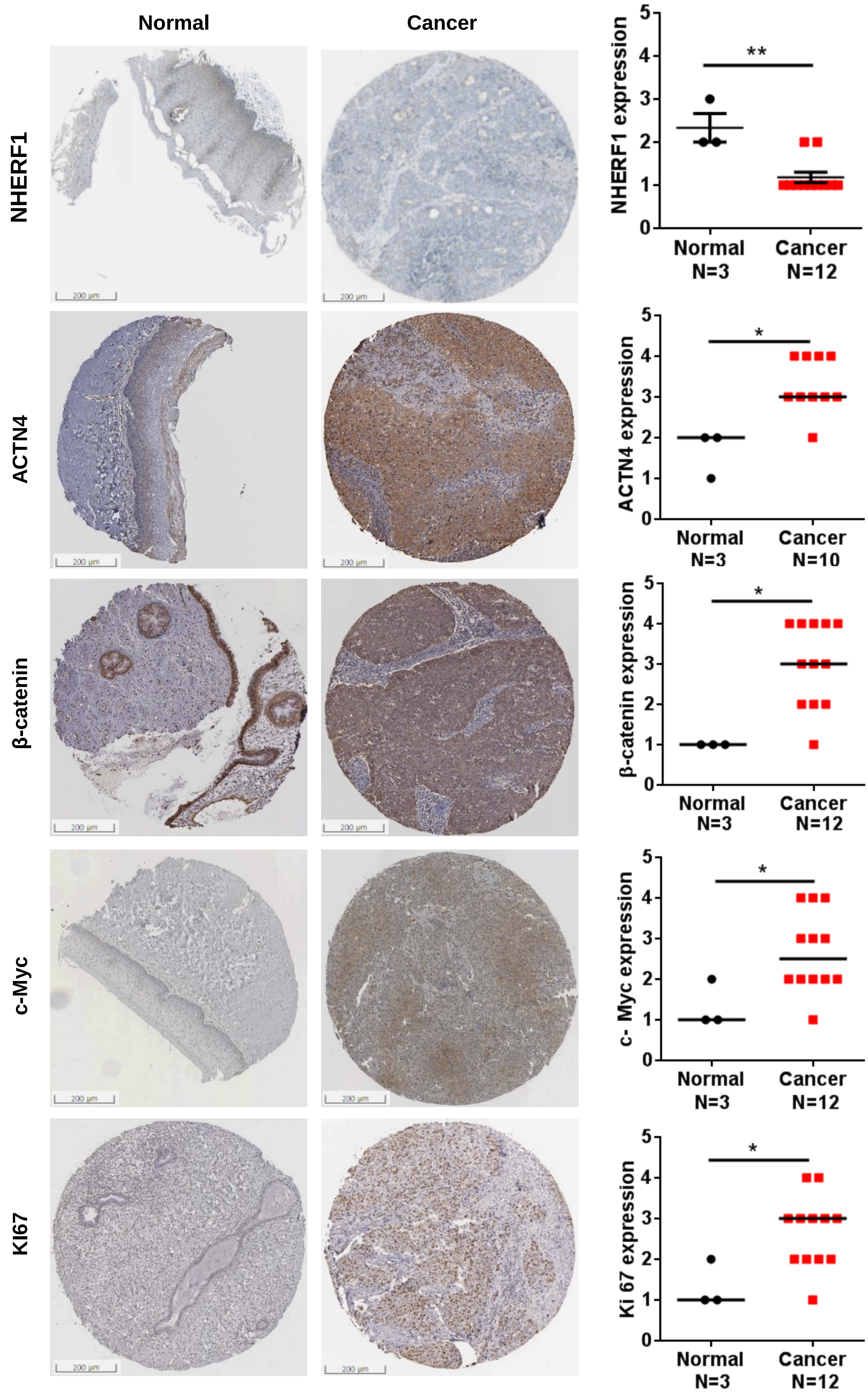

Normal
Cancer
NHERF1
ACTN4
β-catenin
c-Myc
KI67
200 μm
NHERF1 expression
**
Normal N=3
Cancer N=12
ACTN4 expression
*
Normal N=3
Cancer N=10
β-catenin expression
*
Normal N=3
Cancer N=12
c- Myc expression
*
Normal N=3
Cancer N=12
Ki 67 expression
*
Normal N=3
Cancer N=12

Figure S7

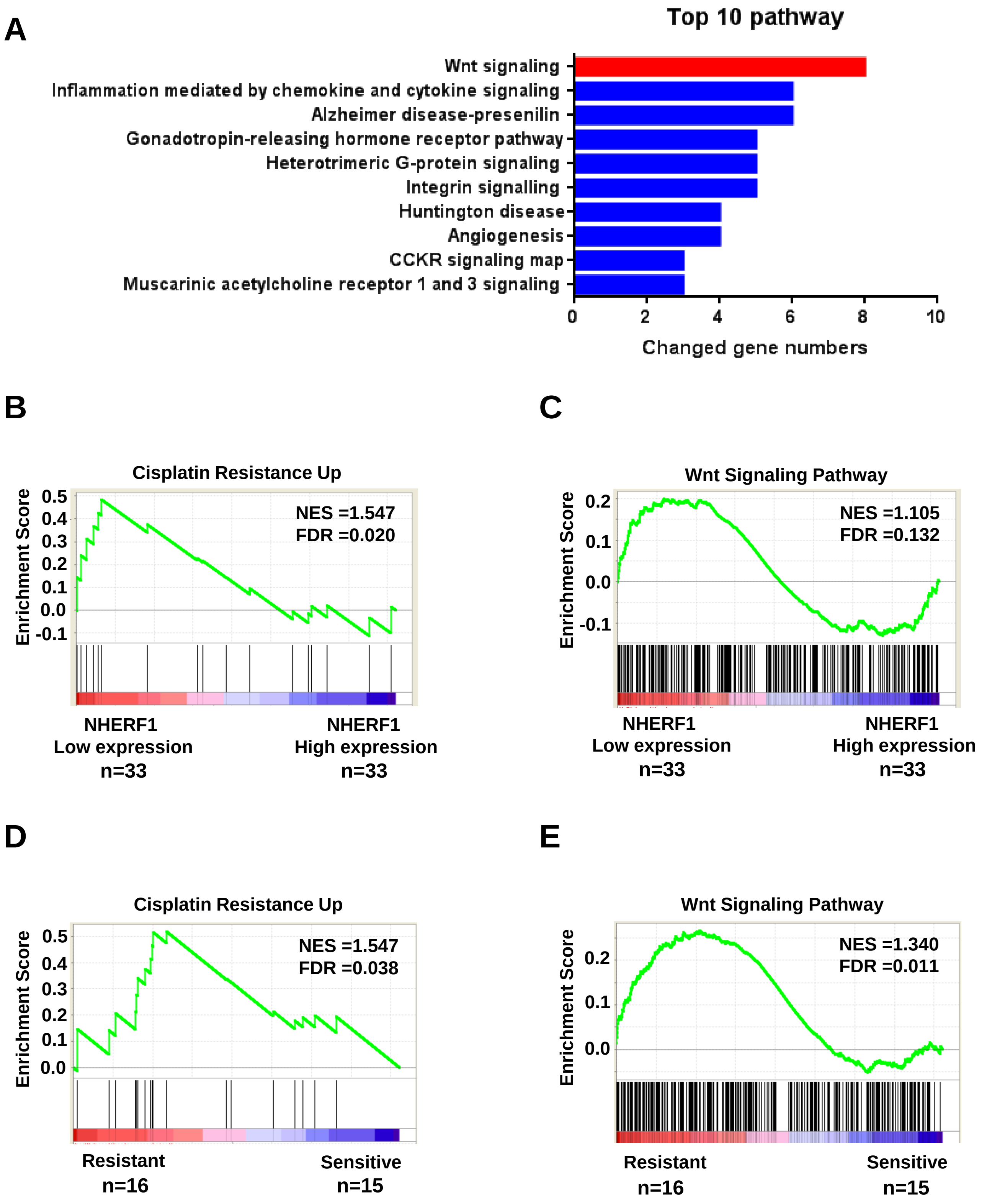

A
Top 10 pathway
Wnt signaling
Inflammation mediated by chemokine and cytokine signaling
Alzheimer disease-presenilin
Gonadotropin-releasing hormone receptor pathway
Heterotrimeric G-protein signaling
Integrin signalling
Huntington disease
Angiogenesis
CCKR signaling map
Muscarinic acetylcholine receptor 1 and 3 signaling
0
2
4
6
8
10
Changed gene numbers
B
Cisplatin Resistance Up
Enrichment Score
0.5
0.4
0.3
0.2
0.1
0.0
-0.1
NES =1.547
FDR =0.020
NHERF1
Low expression
n=33
NHERF1
High expression
n=33
C
Wnt Signaling Pathway
Enrichment Score
0.2
0.1
0.0
-0.1
NES =1.105
FDR =0.132
NHERF1
Low expression
n=33
NHERF1
High expression
n=33
D
Cisplatin Resistance Up
Enrichment Score
0.5
0.4
0.3
0.2
0.1
0.0
NES =1.547
FDR =0.038
Resistant
n=16
Sensitive
n=15
E
Wnt Signaling Pathway
Enrichment Score
0.2
0.1
0.0
NES =1.340
FDR =0.011
Resistant
n=16
Sensitive
n=15

Figure S8

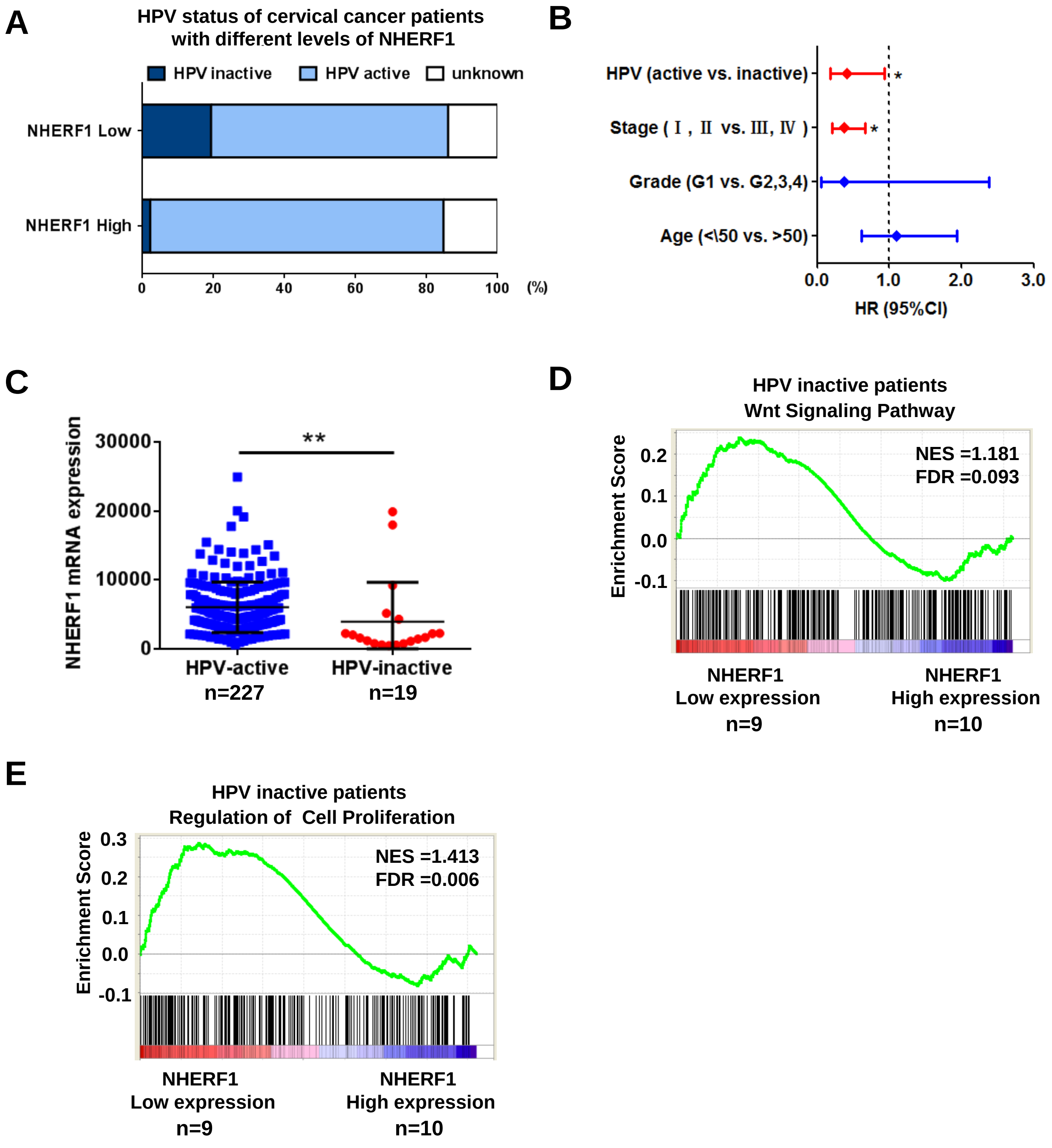
A
HPV status of cervical cancer patients
with different levels of NHERF1
HPV inactive
HPV active
unknown
NHERF1 Low
NHERF1 High
0
20
40
60
80
100
(%)
B
HPV (active vs. inactive)
Stage (Ⅰ , Ⅱ vs. Ⅲ, Ⅳ)
Grade (G1 vs. G2,3,4)
Age (<\50 vs. >50)
*
*
0.0
1.0
2.0
3.0
HR (95%CI)
C
NHERF1 mRNA expression
30000
20000
10000
0
**
HPV-active
n=227
HPV-inactive
n=19
D
HPV inactive patients
Wnt Signaling Pathway
Enrichment Score
0.2
0.1
0.0
-0.1
NES =1.181
FDR =0.093
NHERF1
Low expression
n=9
NHERF1
High expression
n=10
E
HPV inactive patients
Regulation of Cell Proliferation
Enrichment Score
0.3
0.2
0.1
0.0
-0.1
NES =1.413
FDR =0.006
NHERF1
Low expression
n=9
NHERF1
High expression
n=10

Figure S9

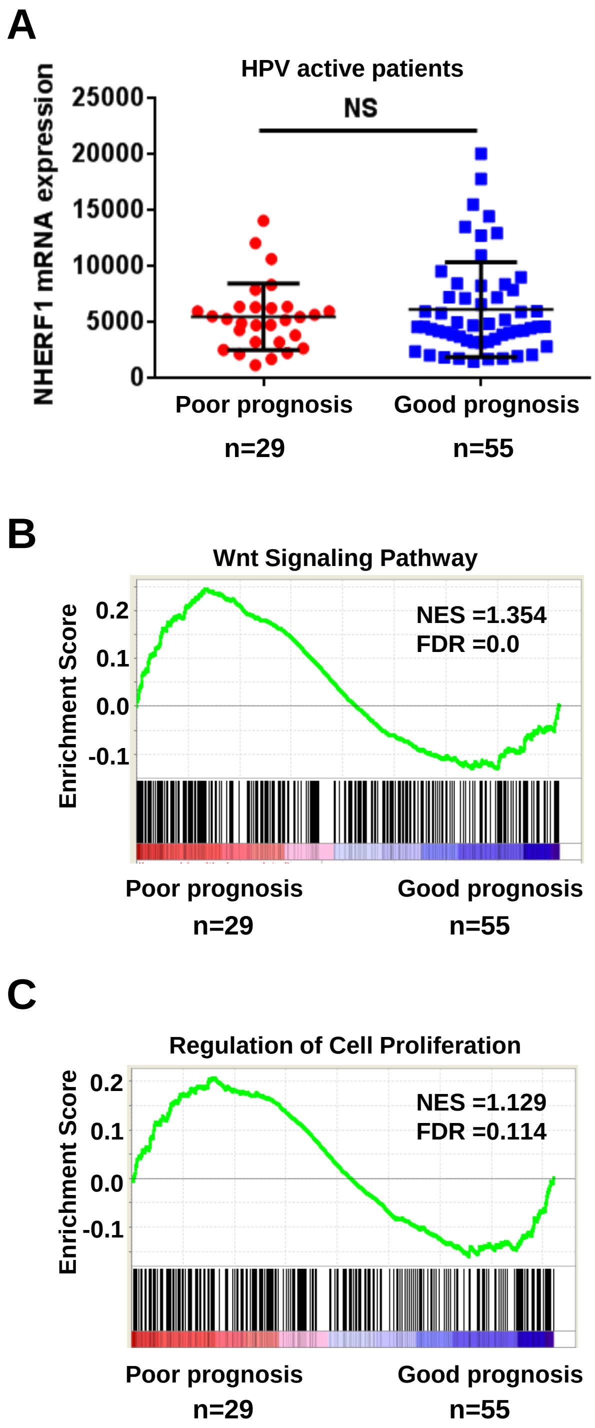
A
HPV active patients
NS
NHERF1 mRNA expression
25000
20000
15000
10000
5000
0
Poor prognosis
Good prognosis
n=29
n=55
B
Wnt Signaling Pathway
Enrichment Score
0.2
0.1
0.0
-0.1
NES =1.354
FDR =0.0
Poor prognosis
Good prognosis
n=29
n=55
C
Regulation of Cell Proliferation
Enrichment Score
0.2
0.1
0.0
-0.1
NES =1.129
FDR =0.114
Poor prognosis
Good prognosis
n=29
n=55
